# Efficacy Assessment of Autodissemination Using Pyriproxyfen-Treated Ovitraps in the Reduction of Dengue Incidence in Parañaque City, Philippines: A Spatial Analysis

**DOI:** 10.3390/tropicalmed8010066

**Published:** 2023-01-16

**Authors:** Antonio D. Ligsay, Zypher Jude G. Regencia, Kristan Jela M. Tambio, Michelle Joyce M. Aytona, Alain Jason A. Generale, Grecebio Jonathan D. Alejandro, Jacquiline S. Tychuaco, Lilian A. De las Llagas, Emmanuel S. Baja, Richard Edward L. Paul

**Affiliations:** 1 The Graduate School, University of Santo Tomas España Blvd., Manila 1008, Philippines; 2 Clinical Research Section, St. Luke’s College of Medicine—William H. Quasha Memorial, 279 E. Rodriguez Sr. Ave, Quezon City 1112, Philippines; 3 Department of Biological Sciences, College of Science, University of Santo Tomas España Blvd., Manila 1008, Philippines; 4 Institute of Clinical Epidemiology, National Institutes of Health, University of the Philippines Manila, 623 Pedro Gil St., Ermita, Manila 1000, Philippines; 5 Department of Clinical Epidemiology, College of Medicine, University of the Philippines Manila, Pedro Gil Street, Taft Ave, Ermita, Manila 1000, Philippines; 6 Department of Biology, College of Science, Polytechnic University of the Philippines, Anonas St., Santa Mesa, Manila 1016, Philippines; 7 Department of Parasitology, College of Public Health, University of the Philippines Manila 625 Pedro Gil St., Ermita, Manila 1000, Philippines; 8 Institut Pasteur, Université de Paris, Centre National de la Recherche Scientifique (CNRS) Unité Mixte de Recherche (UMR) 2000, Ecology and Emergence of Arthropod-Borne Pathogens Unit, 75015 Paris, France

**Keywords:** autodissemination, dengue, ovitraps, Philippines, pyriproxyfen, spatial analysis

## Abstract

Dengue is one of the most important vector-borne diseases worldwide and is a significant public health problem in the tropics. Mosquito control continues to be the primary approach to reducing the disease burden and spread of dengue virus (DENV). Aside from the traditional larviciding and adulticiding interventions, autodissemination using pyriproxyfen-treated (AD-PPF) ovitraps is one of the promising methods to complement existing vector control strategies. Our paper assessed the efficacy of AD-PPF in reducing DENV infections in two barangays in Parañaque City. Using saliva samples from the participants from both the control and intervention sites, we collected the seroprevalence data for three months in each of the two years. Spatial analysis was conducted to determine hotspot areas and identify DENV infection distributions across the trial periods. The results showed that the intervention site was identified as having a clustering of DENV infections in Month 0 of Year 1 and shifted to a random dispersion of dengue cases at the end of Month 3 in Year 2. The disappearance of the clustering of the intervention site translates to a decrease in the cases of DENV infection relative to the control site. Furthermore, we also identified that DENV transmission occurred at a small-scale level that did not go beyond 86 m. In conclusion, AD-PPF is suggested to be an effective strategy and may be used as an additional vector control approach, albeit based on this short-term implementation.

## 1. Introduction

Dengue virus (DENV) is a Flavivirus, part of the *Flaviviridae* family, with a single strand RNA and includes four viral serotypes (DENV-1, DENV-2, DENV-3, DENV-4) [1]. DENV infection can cause serious medical problems, such as dengue hemorrhagic fever (DHF) and dengue shock syndrome [2], which can lead to the death of the infected individual. Severe dengue was first reported in the 1950s during dengue outbreaks in the Philippines [3]. DENV is vectored by *Aedes* spp. mosquitoes, primarily *Aedes aegypti,* in tropical countries. Climatic factors such as precipitation, humidity, and temperature impact mosquito development and survival; increased rainfall provides many breeding grounds for mosquito larvae, such as pools of water, and humidity influences adult mosquito survival and biting rate [4]. Changes in these conditions affect DENV transmission rates with subsequent consequences for the health burden [5]. 

Over the last few decades, there has been a dramatic increase in dengue cases, globally, and in both urban and rural areas of the Philippine archipelago [6]. Dengue fever has become a significant health threat in subtropical countries, particularly the Philippines, where dengue is endemic and has been an increasing cause of hospitalization and death in recent years [7,8,9]. The incidence of dengue in the Philippines increased from 37,101 in 2006 to 118,868 in 2010 [10]. Significant outbreaks since 2012 recorded 187,000 dengue cases and as many as 270,00 cases between January and August 2019 [11], ranking the country fourth out of ten in ASEAN [12]. The degradation of the environment due to urbanization, an increase in population, poor waste disposal strategies, changes in weather conditions, and poor surveillance of mosquito breeding sites are among the factors that contribute to the rise in dengue cases in the country [13,14]. 

Without vaccines and pharmacological treatment, mosquito control and awareness campaigns are the only measures to limit transmission [15]. Community involvement is vital in addressing the control of dengue. Successful interventions can be achieved with education campaigns and full co-operation between members of society, which offers an excellent strategy to eliminate the source of the disease [16]. Recently, to respond to the escalating burden of dengue, the WHO attempted to galvanize mosquito-control strategies through an integrated vector management plan [17,18]. However, a suite of intervention methods that can easily be implemented across the epidemiological contexts that arboviruses inhabit remains a nagging issue in the control of dengue. The most feasible strategy is to address mosquito breeding sites to stop their reproduction [19] and therefore lessen the population of mosquitoes. The development and validation of alternative methods are critical, particularly in the urban setting, as they may not only have broad utility but can be implemented per specific epidemiological situations [20].

One of the alternative ways is to control the breeding sites of the vector at its larval stage (larviciding) [21]. Whilst larviciding can be effective, it is laborious and may fail to reach the cryptic aquatic habitats used by mosquitoes for oviposition. One promising approach in larviciding is to exploit the ‘skip-oviposition’ behavior of adult female *Ae. aegypti* to disseminate insecticide to natural larval habitats following exposure to a contaminated surface [22], a technique known as autodissemination (AD). The concept of AD is not new, as it was previously used in agriculture [23]. One of the most prominent examples was the use of biocontrol agents in honeybees during the 1990s. The use of AD was found to be more efficient than conventional sprayers to spread the inoculums against a pest-infested flower [24]. This concept was later adapted to control the mosquitoes that transmit DENV [25]. This approach has been shown to be effective in reducing mosquito densities in several field trials [26,27,28] through the use of pyriproxyfen (PPF), a synthetic analog of a juvenile growth hormone that has high potency at very low concentrations [29]. Most of these studies showed that AD occurred and successfully reduced the *Aedes* spp. populations. The fundamental underlying feature of using PPF is its high potency at nanogram concentrations enabling mosquitoes to carry it and contaminate nearby aquatic habitats and control mosquitoes across the whole area [30].

Although AD has shown entomological efficacy in some countries and can be considered as an additional approach in an existing vector control program, measures of its actual epidemiological impact (infection reduction) are generally inconclusive [31,32]. Tsunado et al. implemented a small-scale prospective trial, implementing Olyset^®^Net plus pyriproxyfen in water containers with follow-up once a year for two years to assess the sero-conversion [33]. No epidemiological impact was observed. Ocampo et al. carried out a community-based intervention by distributing pyriproxyfen in street catch basins and compared the dengue incidence reported to the health authorities with that from an untreated neighboring town [34]. A reduction in disease incidence was observed in the treated town. Our study aimed to assess the epidemiological efficacy of AD-PPF in reducing dengue incidence using a reactive trap deployment strategy around dengue index cases and implementing spatial analysis. 

## 2. Materials and Methods

### 2.1. Study Design, Sites, and Population

A quasi-experimental study design was used in this study, wherein two groups were formed: the control and the intervention groups. The Department of Health (DOH)—Metro Manila Centers for Health Development data on the dengue incidence were obtained. The National Capital Region cities with high rates of dengue attacks were selected for the study. Parañaque City in the south of Manila, with an approximate population of 0.5 million [35], was chosen. The criteria for choosing barangay sites in Parañaque City involved areas that constantly reported 10–50 dengue cases on a monthly basis and with repeated hotspots during the low transmission months. Barangays Sto. Niño (120°59′45″–120°59′54″ East; 14°30′18″–14°30′32″ North) and San Dionisio (120°59′11″–120°59′21″ East; 14°29′1″–14°29′19″ North) were selected as the control and intervention groups, respectively. These barangays are in adjacent proximity to one another, but are not juxtaposed. The control and the intervention areas were matched as carefully as possible according to the data on the local dengue incidence. Once a dengue index case was reported within a site, ovitraps (mosquito density monitoring devices) were installed for two weeks. The installation was conducted to evaluate whether mosquito densities (>5% ovitraps positive for mosquito larvae) were sufficiently high for active DENV transmission and, therefore, selectable as a study site.

#### 2.1.1. Participant Inclusion Criteria

After selecting the control and intervention sites, we invited participants residing within 200 m of the dengue index case. Invited participants were all within the age group of 1–30 years old, willing to participate in the study, and could provide saliva samples. The highest incidence of dengue is among children and adults aged 1–30 years old [36]; hence, they are the population of interest. 

#### 2.1.2. Participant Exclusion Criteria

Participants who were suffering from other chronic diseases and were febrile and/or refused to sign the informed consent form were excluded at the time of data collection.

### 2.2. Data Collection

#### 2.2.1. Control and Intervention Ovitrap Set-Up

Co-ordination with the Local Government Unit (LGU), DOH—Rural Health Units, and the Department of Education was conducted to achieve the smooth implementation of the project. Individuals recruited for seroconversion were those within 200 m of the household of the index dengue case and within 10 m of the autodissemination and ovitrap stations. At the time of recruitment, AD-PPF devices (Ovitraps filled with 250 mL of water with one sachet of Sumilarv™ 0.5 G Sumitomo Chemical as previously described elsewhere [28]) were placed in the chosen households in the intervention study site houses. In addition, traps without the PPF mixture were similarly deployed in the control site. One trap was placed per household. The barangay health workers inspected the ovitraps every week, and the contents of all of the ovitraps were serviced every two weeks. If the ovitraps’ contents were observed to be less than the required volume (half-full), the ovitraps (with or without PPF) were refilled.

#### 2.2.2. Outcome Measurements

Once an index case was detected in the study sites, saliva specimens were collected from a random sample of children and young adults and consenting household members of houses neighboring the index case location to determine antibody levels (Immunoglobulin G, IgG). There were three paired samples from each individual over the 3 month trial period in each year. Information on the Global Positioning System (GPS) position of the household members and neighbors was gathered, and a code was assigned to each individual and to the saliva samples. Saliva specimens were collected on a monthly interval from August to November, representing the third quarter cold-wet seasons of each year (2017 and 2018). After the last collection month, IgG determination was conducted using an enzyme-linked immunoassay (ELISA), following the previously described procedures [37]. Positive and negative controls previously confirmed as control references were provided by the Institut Pasteur Cambodia [38]. An optical density (OD) difference between the sample and the control, of 0.1 and above, was considered positive for IgG. We analyzed all of the saliva samples of each participant from Months 1 to 3, simultaneously.

#### 2.2.3. Sample Size Calculations

The sample size was calculated using the following standard formula when considering proportions (incidence rate): n = 2(Za + Z1 – β)^2^/p(1 – p)/(p0 – p1)^2^, where n is the required sample size per treatment arm (control or treatment), Zα and Z1–β are the constants set by the convention according to the accepted α error and whether a one-sided or two-sided effect, p0 is the proportion infected in control areas, and p1 the proportion infected in treatment areas (p = (p0 + p1)/2). Assuming a *p* < 0.05 as acceptable and a study with 90% power, the following constant values are: Zα = 1.96 (two-tailed); Z1 − β = 1.2816. The final sample size will depend on the dengue transmission rate in the study areas as estimated from the historical mean rate. The historical annual incidence rates were estimated at between 11 and 22% [36]; thus, an estimated incidence rate (p0) of 10% was chosen. For an efficacy of treatment of 40% (i.e., reduction in dengue), with 90% power, a minimum of 700 individuals per area would need to be recruited in the treatment site, with an equivalent number in the control site. 

### 2.3. Data Analysis

Using ArcGIS Pro 2.9.1 (ESRI, Redlands, CA, USA), a software that allows for the handling and analyzing of geographic information by visualizing geographical statistics through layer building maps, the co-ordinates of each recorded result were georeferenced with the world street map as the base map [39]. The maps were generated for the categorized data monthly and the aggregated data per year.

#### 2.3.1. Spatial Statistics

We utilized the statistical tools in the spatial statistics toolbox. This toolbox contained toolsets for analyzing spatial distributions, patterns, processes, and relationships. Two of these toolsets were used in this study. First, the analyzing patterns toolset was used to evaluate whether the features or the values associated with the features form a spatial pattern (i.e., clustered, dispersed, or random). Second, the mapping clusters toolset was used to identify statistically significant hot or cold spots [40,41,42].

#### 2.3.2. Analyzing Patterns

The analyzing patterns tools are inferential statistics that provide statistics to quantify the broad patterns observed in the data feature, particularly identified as clustered, dispersed, or random spatial patterns [40,42]. This tool calculated the Moran’s I Index value and both a z-score and *p*-value to evaluate the significance of that Index [43]. The detailed calculation of the Global Moran’s I statistic followed Equation (1) below:

(1) $$I=\frac{n}{S_{0}}\frac{\sum_{i=1}^{n}\sum_{j=1}^{n}w_{i,j}z_{i}z_{j}}{\sum_{i=1}^{n}z_{i}^{2}}$$

The *z_i_* is the deviation of an attribute for feature *i* from its mean (*x*_1_ − *X*), *w_i,j_* is the spatial weight between *i* and *j*, n is equal to the total number of features, and S_0_ is the aggregate of all special weights following Equation (2).

(2) $$S_{0}=\overset{n}{\underset{i=1}{\sum }}\overset{n}{\underset{j=1}{\sum }}w_{i,j}$$

#### 2.3.3. Mapping Clusters

The tools in the mapping clusters performed the analysis to identify the locations of statistically significant hotspots, coldspots, spatial outliers, and similar features/zones. In this study, the optimized hotspot analysis tool was employed. This tool identifies statistically significant spatial clusters of high values (hotspots) and low values (coldspots) [40]. The input features used in this analysis were the point feature of each serostatus result. In addition, the analysis field containing the incident counts to serve as the values to be analyzed is the aggregated dengue count per year (i.e., 0, 1, 2, and 3).

Furthermore, the scale of analysis was set to the default optimal distance computed in the statistics of analysis. Given the incident points or weighted features (points or polygons), this tool created a map of statistically significant hotspots and coldspots using the Getis-Ord Gi* statistic. Using this statistic, we evaluated the characteristics of the input feature class to produce optimal results. Moreover, it automatically aggregated the incident data, identified an appropriate scale of analysis, and corrected for multiple testing and spatial dependence. In addition, we interrogated the data to determine the settings that would produce optimal hotspot analysis results [39,44].

## 3. Results

A total of 1462 individuals participated in either the intervention (San Dionisio, n = 705) or control groups (Sto. Niño, n = 757). Most of the participants from both sites were females and belonged to the 1 to 10 years age group (see Table 1 for details).

A total of 672 individuals were tested for serostatus in San Dionisio during Year 1, while a total of 577 individuals were tested in Year 2. In Sto. Niño, 745 individuals were tested for serostatus in Year 1, and 637 were tested in Year 2. The highest positive dengue count in San Dionisio was recorded during Month 3 of Year 1 (103 cases), while Sto. Niño recorded the highest positive dengue count at 15 cases during Month 1 of Years 1 and 2. Taking the monthly average per year, Year 1 of San Dionisio had a positive dengue count of 81 cases, while Year 2 had 11 cases. In contrast, Sto. Niño had a monthly average of positive dengue count of 11 and 13 cases for Years 1 and 2, respectively. See Table 2 for the monthly count of the positive and negative serostatus results.

### 3.1. Map Generation

**Figure 1:**
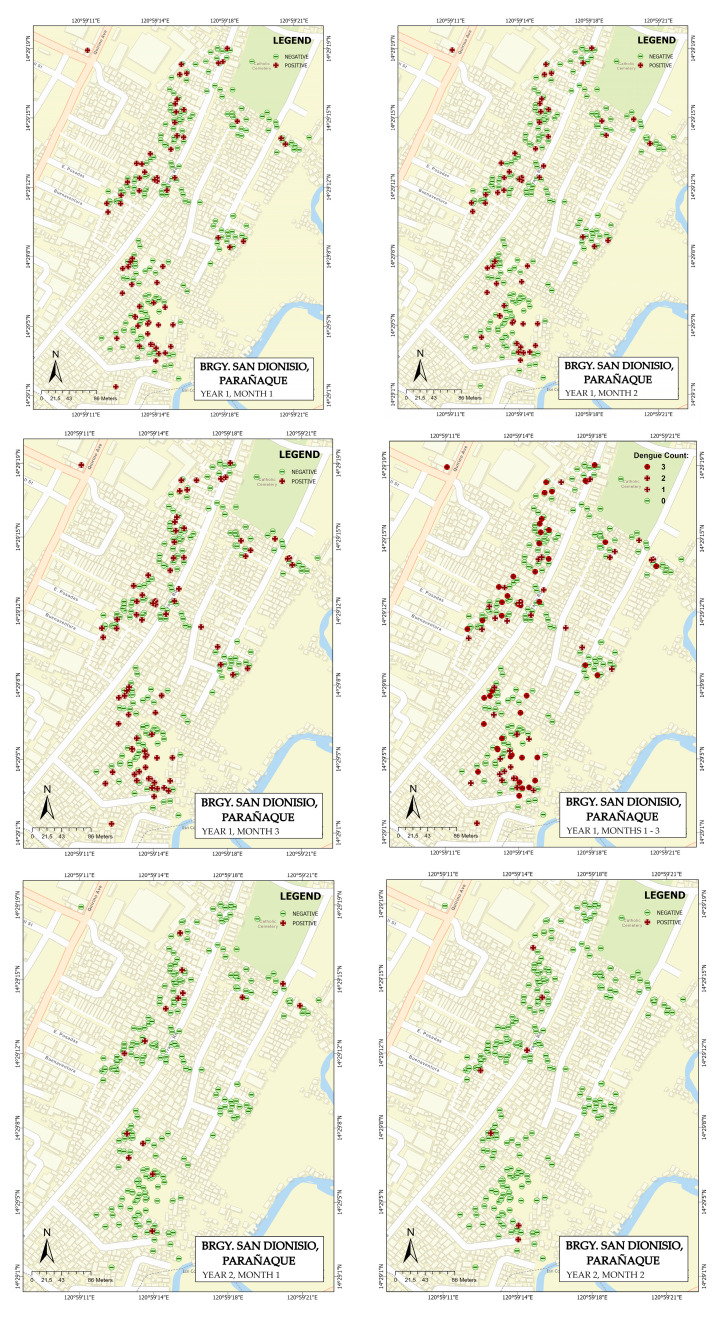

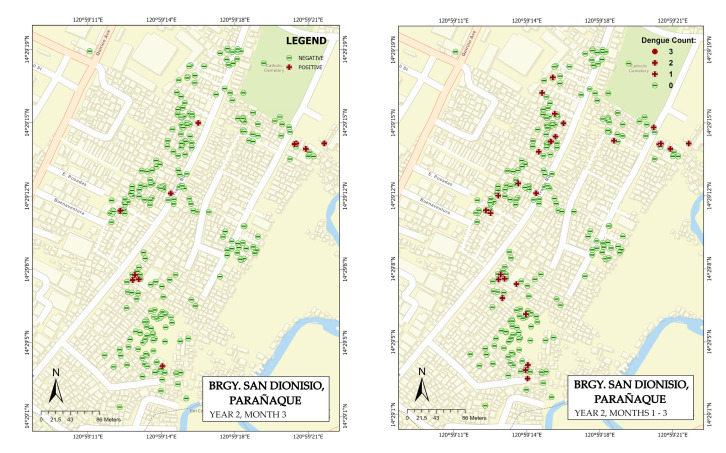
Generated maps of the San Dionisio site display both the positive and negative serostatus test results, monthly and yearly.

**Figure 2:**
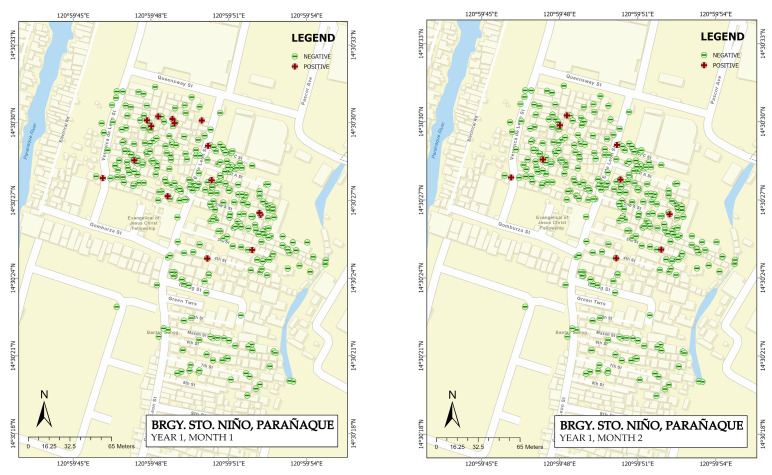

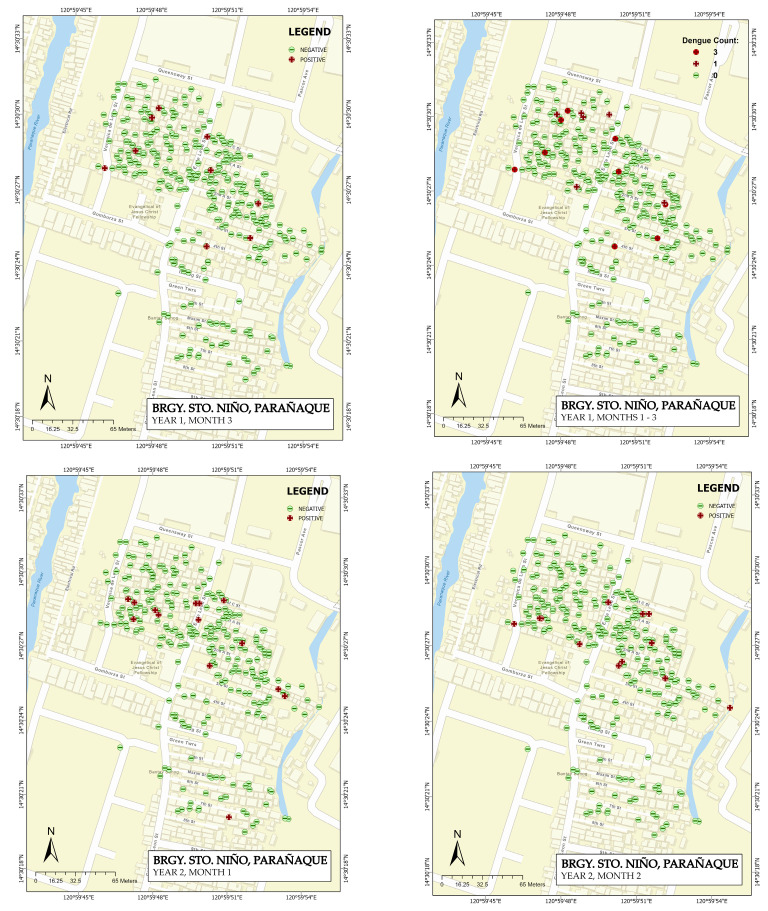

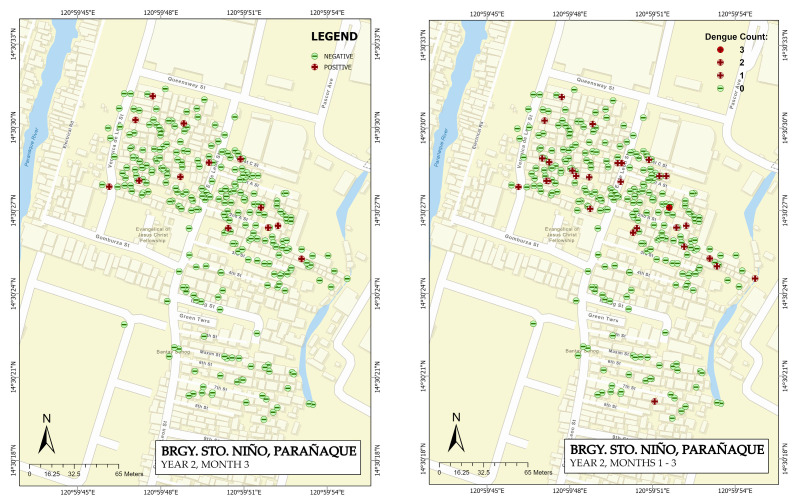
Generated maps of the Sto. Niño site display both the positive and negative serostatus test results, monthly and yearly.

The recorded dengue cases for barangays San Dionisio and barangay Sto. Niño were mapped using ArcGIS Pro 2.9.1. The maps were generated per month, displaying the positive and negative serostatus test results. Figure 1 shows the generated maps for San Dionisio, while the maps for Sto. Niño are displayed in Figure 2. The yearly aggregate count was used to proceed with the spatial analysis using the spatial statistics tools available in ArcGIS Pro 2.9.1.

### 3.2. Global Moran’s I Spatial Autocorrelation

**Figure 3:**
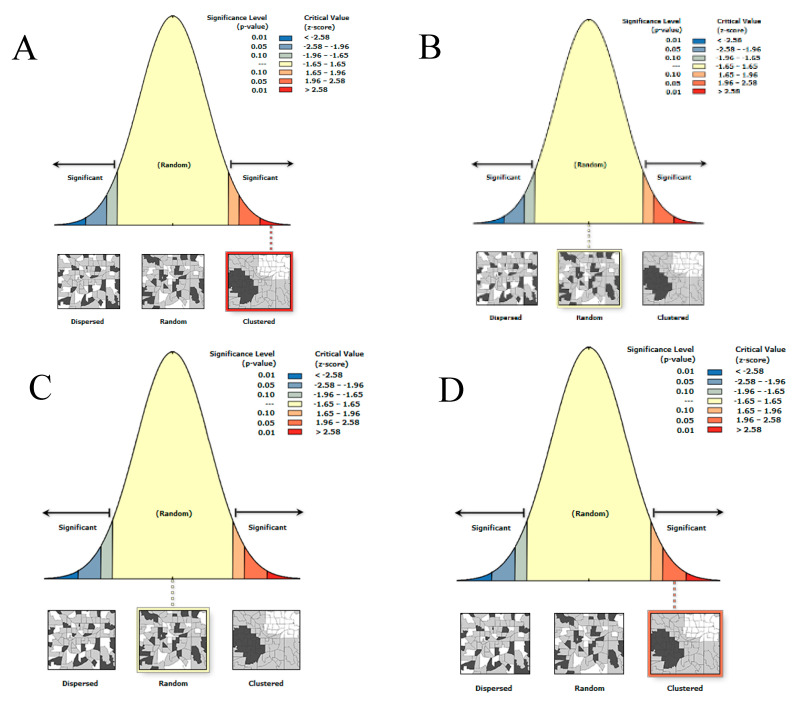
Global Moran’s I spatial autocorrelation result: San Dionisio Year 1 (**A**) and Year 2 (**B**), and Sto. Niño Year 1 (**C**) and Year 2 (**D**).

Table 3 and Figure 3 present the result of the Global Moran’s I spatial autocorrelation statistics. The results can be interpreted within the context of its null hypothesis by looking at the *p*-value and z-score. A statistically significant *p*-value (≥0.05) with a positive z-score signifies that the data are in a random pattern, shown from San Dionisio in Year 2 and Sto Nino in Year 1. An insignificant *p*-value (≤0.05) indicates that the data are in a clustered pattern, shown from San Dionisio in Year 1 and Sto. Nino in Year 2.

### 3.3. Optimized Hotspot Analysis of Clustered Years

**Figure 4:**
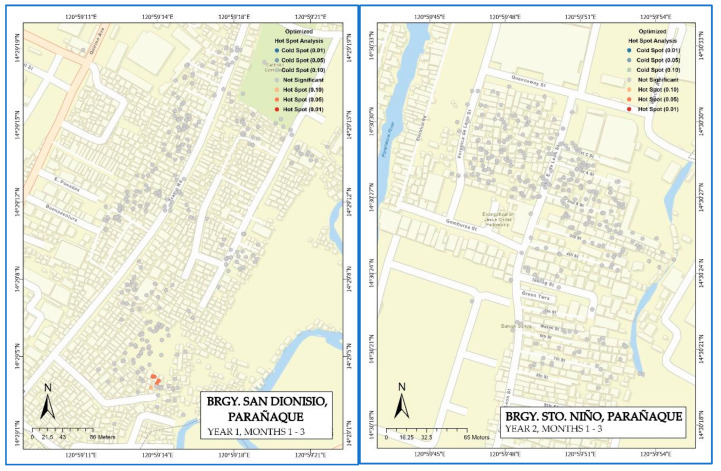
Optimized Hot Spot Analysis map for San Dionisio (year 1) and Sto. Niño (Year 2).

A total of 660 valid data points in San Dionisio (Year 1) were included (Table 4). With a minimum count of 0 and a maximum of 3 dengue counts, each point feature had an average of 0.3682 dengue counts and a standard deviation of 0.8974. There were three locational outliers detected, and the optimal fixed distance band computed was 30 m. The optimized hot spot analysis detected ten statistically significant output features with these parameters. Figure 4 presents the optimized hot spot analysis map. Eight output features were the identified hot spots at a 5% significance level, while the other two were at a 10% significance level.

Sto. Niño (Year 2) had 640 valid input features (Table 4). With a minimum count of 0 and a maximum of 3 dengue counts, each point feature has an average of 0.0625 dengue count and a standard deviation of 0.2945. There were two locational outliers detected, and the optimal fixed distance band computed was 84.7478 m. With these parameters, no output features were detected to be statistically significant using the optimized hot spot analysis. The map for this analysis is also displayed in Figure 4.

## 4. Discussion

Before the Coronavirus-2019 (COVID-19) pandemic, dengue substantially burdened communities and health systems in most tropical and subtropical countries, including the Philippines [45]. One of the most effective ways to reduce dengue cases is to manage the vector that carries the DENV. The use of AD-PPF as a strategy to control mosquito density has been proven effective based on previous research [46,47,48]. In the Philippines, previous research has tackled the use of AD-PPF in a small urban community in Metro Manila using a small sample size; the results of this study showed that dengue seroconversion rates decreased in the treated population, but not significantly so. The authors hypothesized that the use of PPF-treated ovitraps as a reactive strategy to the identification of a dengue case may have impacted the mosquito population, but not the seroconversion rates, it being too late to stop the transmission of that dengue case [28]. Our study, which examined the pattern of the spatial distribution of DENV infections in two research sites in Parañaque City, may have rectified the limitations seen from the previous AD-PPF research. Although dengue sero-positivity remained relatively low in the control site over both years, it dramatically decreased in the intervention site in the second year. This would suggest that the intervention successfully contained the epidemic potential and prevented it from expanding further. Whilst herd immunity may have contributed to lessening the number of susceptible individuals locally, the numbers of sero-negatives were still the majority at the end of Month 3 in the epidemic year and should have provided a significant source of susceptible individuals. This suggests that the intervention may have been effective and that a reactive strategy may work.

A Geographic Information System (GIS) was implemented to visually assess the distribution of DENV infections in the chosen communities. Using this information, spatial analysis was carried out to determine the distribution patterns of DENV infections in each research site. The emergence of spatial epidemiological research on various intervention and non-intervention studies has a long history. However, research tackling the use of GIS is a recently emerging field of study. With the advancement in modern technology and progress in spatial analysis, the use of GIS in health research has become of utmost importance [49,50,51,52]. To control inevitable disease outbreaks, intensive monitoring and planning strategies for epidemics, including dengue, are very important. Our paper aimed to give insights into the use of GIS and spatial pattern analysis on a specific dengue control measure, which is the use of AD-PPF in a small urban community. Spatial autocorrelation analysis also proved to be a valuable tool for analyzing the spatial patterns that change over time. Our study demonstrated that the spatial distribution patterns of dengue incidence were significantly clustered and identified the dengue hotspots in the early phase of the intervention site and the latter part of the control site. After continuous exposure to AD-PPF, the dengue incidence in the intervention site shifted from clustering to random dispersion. This shift in the dispersion of DENV infection or the disappearance of DENV infection clustering in the intervention signifies that there was a decrease in the transmission rate of dengue infection in this densely populated area. In addition, the use of GIS to assess the spatial patterns of DENV infections is relatively new. It has been previously studied in Asian countries, such as Pakistan [53], India [54], China [55], and other Southeast Asian countries [56,57,58]. However, our study, which utilized spatial analysis to assess the efficacy of AD-PPF by determining the clustering of dengue incidence, is new. Previous studies on AD-PPF only involved the evaluation of the efficacy of PPF granules [34,59,60] or the assessment of *Aedes* mosquitoes’ resistance to PPF [61,62,63], but not on the use of spatial analysis to identify the clustering of DENV infection after being exposed to AD-PPF.

The hotspots or clustering observed after the optimized hotspot analysis indicated that most of the clustering distance did not extend beyond 86 m, which may suggest that an underlying spatial process of dengue transmission is acting at such a small scale. The DENV infection transmission in our areas of study was highly focal (radius = 10 to 200 m). This may suggest that the critical parameters needed for the oviposition, growth, feeding, and reproduction of the *Aedes* vector were present at the domestic level. Congruent with our study results, previous research reported a short flight range for *Ae. aegypti* (radius < 40 m), where the mosquito tends to be spatially clustered at the household level in relation to the occurrence of indoor breeding sites and abundant human hosts [64,65]. Conditions that increased the risk of DENV infection transmission and generated a hotspot may reflect a number of underlying local features of the environment, including local urban heat islands, water storage, and high human densities, amongst other recognized risk factors associated with increased mosquito vectorial capacity [66,67,68,69].

Urbanization and the rapid movement of humans are also essential factors in the dynamics of DENV infection transmission [14]. Compared with the younger population, the adults are generally more mobile; hence, the younger population who stay at home are more at risk of DENV infection due to the presence of mosquitoes in the community [70]. In addition, the interactions among each of the participants in the neighborhood should not be dismissed and may have influenced the increase in DENV infection transmission, hence the observed clustering. Future studies on the spatial analysis of dengue incidence may look into the possible reasons for clustering as this may provide helpful information on specific ways to mitigate DENV infection. Moreover, it is essential to determine the factors underlying the clustering and how the clustering changes over time. Techniques using spatial analysis applied to vector-borne diseases such as DENV infection have been proven effective [58,71], similar to that demonstrated in our study. These techniques determine the high-risk transmission areas, and the information generated may inform health authorities on better-targeted dengue control measures and surveillance. 

Our study also provided helpful information on seroconversion detection by measuring the IgG from the participants’ saliva samples. The use of saliva, rather than serum samples, as an alternative method to detect DENV infection has been reported to be effective and innovative in previous research on dengue [35,72]. 

To the best of our knowledge, this is the first study in the Philippines to assess the efficacy of AD-PPF implementation to reduce DENV infection by analyzing the spatial cases of dengue incidence in a specific period. This study provides the basis for future investigations into the environmental or social factors influencing the changing dengue case patterns. The results of our study may be helpful to complement the current strategies in controlling vector-borne disease in the Philippines, as our study results indicate the efficacy of AD-PPF. Reactive strategies around dengue cases are more in line with the current public health practices of fumigating around dengue cases. The use of AD-PPF traps may enable a longer-term impact around dengue cases with little extra effort. Moreover, the results of our study may be used to determine areas at risk for DENV infection. With the use of saliva samples to diagnose DENV infection and the use of spatial analysis, dengue surveillance strategies may be developed in areas where AD-PPF and other public health interventions are needed. 

Our study has several limitations, including the short-term exposure to AD-PPF and the non-inclusion of different climactic factors. However, the two sites were geographically very close and thus likely to have experienced the same climate. Despite the short-term use of AD-PPF, the study results are promising in reducing dengue incidence. Nevertheless, due to its short implementation in the selected areas in a poor urban community, it is recommended to scale up the implementation of PPF-treated ovitrap in all houses, and not only in designated sites, to capture all possible geographical parameters, particularly given the short flight distance of mosquitoes and, hence, the short distance spread of PPF, as found previously [27]. Moreover, intensive community-based campaigns on the use of PPF-treated ovitraps are also recommended. Integrating community or barangay leaders and other stakeholders, such as health officials, in efforts to eliminate container mosquito larval habitats is crucial to the success and sustainability of this community-based intervention program. In addition, community engagement in health education campaigns should complement this approach.

Furthermore, AD-PPF trials should be implemented countrywide to capture all types of climatic conditions in the country. Through workshops, scaling up and the increased implementation of this ovitrap approach to other regions in the Philippines, it is possible that a training and certification program could be developed. Such a certification would create a network of public health individuals who can address container mosquito control within their communities. Empowering communities to lead this approach is critical to the sustainability of such a program, particularly in the Philippines, where geographical features are a challenge. In addition, our study only focused on the variation of DENV clustering after AD-PPF implementation, but did not examine its causes. Future research should determine the different socio-environmental key factors (e.g., social, demographic, climate, vegetation, and mosquito density) which may affect the transmission patterns of DENV. 

## 5. Conclusions

We demonstrated the application of GIS and the use of spatial analysis to detect DENV infection transmission in areas exposed to AD-PPF as an intervention to decrease dengue incidence. Using saliva samples collected from the participants from both control and intervention sites in Parañaque City, we gathered seroprevalence data for three months in two years and assessed the efficacy of AD-PPF. The intervention site was identified as having a clustering of DENV infections in Month 0 of Year 1 and shifted to a random dispersion of dengue cases at the end of Month 3 in Year 2. The shift in the spatial distribution of DENV infections across the intervention site that was not seen in the control area implies that the AD-PPF implementation decreased dengue incidence over a specific period. Our collected seroprevalence data also included asymptomatic and symptomatic infections, which provided a clearer view of the high-transmission risk areas. We also identified that the hotspots clustered in one area of the site and that the DENV infection transmission occurs at a domestic level (radius = 10 to 200 m). The results of our study provided evidence that, albeit with short-term exposure to AD-PPF, dengue incidence can be reduced, and with a tool that aligns with the current public health strategy of reacting to dengue cases. Furthermore, the use of GIS and spatial analysis to identify hotspots of dengue transmission are essential tools to create targeted and improved public health interventions in areas where DENV infection is rampant.

## Figures and Tables

**Table 1:** Characteristics of the study population.

Characteristics ^a^	Barangays	
	San Dionisio (n = 705)	Sto. Niño (n = 757)
Age, years	9.9 ± 5.6	13.1 ± 7.4
Age categories, years		
1 to 10	61.7	43.0
11 to 20	33.2	40.3
21 to 30	5.1	16.7
Male	48.8	48.3

^a^ Distributions of variables are reported as percentages or mean ± standard deviation.

**Table 2:** Monthly count of the positive and negative serostatus results in barangays San Dionisio and Sto. Niño for Years 1 and 2.

Barangays	Collection Dates		Serostatus	
			Positive	Negative
San Dionisio	Year 1	Month 1	76	598
		Month 2	65	609
		Month 3	103	566
	Year 2	Month 1	16	561
		Month 2	7	570
		Month 3	11	567
Sto. Niño	Year 1	Month 1	15	730
		Month 2	9	736
		Month 3	9	736
	Year 2	Month 1	15	630
		Month 2	13	620
		Month 3	13	620

**Table 3:** Summary of the Global Moran’s I spatial autocorrelation results in San Dionisio and Sto. Niño.

Barangays	Date	Moran’s Index	z-Scores	*p*-Values	Interpretation
San Dionisio	Year 1	0.027112	2.74	0.01	clustered
	Year 2	0.004152	0.51	0.61	random
Sto. Niño	Year 1	−0.00263	−0.18	0.86	random
	Year 2	0.017973	2.35	0.02	clustered

**Table 4:** Optimized hotspot analysis results summary in San Dionisio (Year 1) and Sto. Niño (Year 2).

Parameters	San Dionisio, Year 1	Sto. Niño, Year 2
Initial Data Assessment	660 input features	640 input features
Data Summary		
Minimum count	0	0
Maximum count	3	3
Mean count	0.3682	0.0625
Standard Deviation	0.8974	0.2945
Locational Outliers	3 outlier locations	2 outlier locations
Scale of Analysis	30 m optimal distance	85 m optimal distance
Hotspot Analysis	10 output features	0 output features

## Data Availability

The authors confirm that some access restrictions apply to the data. The researchers interested in using the data must obtain approval from the St. Luke’s Medical Center’s College of Medicine Research Ethics Committee. The researchers using the data are required to follow the terms of a number of clauses designed to ensure the protection of privacy and compliance with the relevant Data Privacy Act of the Philippines. Data requests may be subject to further review by the Ethics Committee and may also be subject to individual participant consent.
